# Editorial: Exploring microbial mat communities in extreme environments

**DOI:** 10.3389/fmicb.2024.1440619

**Published:** 2024-06-25

**Authors:** Ian Hawes, José Q. García-Maldonado, Luisa I. Falcón

**Affiliations:** ^1^Coastal Marine Field Station, University of Waikato, Tauranga, New Zealand; ^2^Centro de Investigación y de Estudios Avanzados del Instituto Politécnico Nacional, Unidad Mérida, Yucatan, Mexico; ^3^Universidad Nacional Autónoma de México (UNAM), Instituto de Ecología, Unidad Mérida, Yucatan, Mexico

**Keywords:** microbial mats, extreme environments, Antarctica, cyanobacteria, microbial ecology

Microbial mats are biodiverse communities within which multiple functional groups of microbes combine to capture, sequester and recycle nutrients and energy. They are one of the earliest known and organized communities in the Earth's fossil record when they were widespread and abundant for billions of years. Today they are best developed in extreme environments that exclude larger metazoans which disturb mat development in more benign habitats. Microbial mats and extreme environments are thus intimately linked, and provide great opportunities to study diversity, evolutionary processes and the capacity of life to adapt to environmental extremes. The aim of this Research Topic was to look across a range of environments, from very hot to very cold, hypersaline to hyper arid, tropical to polar, to contribute to our collective understanding of the diversity, complexity, and ecological significance of microbial mats. Within this topic, eight articles have been published that extend our knowledge of the diversity and interactions of microbial mats in extreme environments and explore the limits of survival and the mechanisms that enable life to persist at the edge of the habitable envelope.

Almela et al., analyzed the effect of nutrient inputs from macrofauna on microbial mats in Antarctica following a coupled approach including 16S rRNA gene sequencing, stable isotopes, and nutrient level analysis, and recognized the effect of penguin colonies and other marine vertebrates on microbial mat community composition.

Han et al., studied the archaeal and bacterial composition of a sediment core from the Chuckchi Shelf off the western Arctic Ocean following a metabarcoding-based sequencing and qPCR. This study proposes the relevance of bacterial diversity specifically in the sulfate–methane transition zone, while recognizing past cyanobacterial blooms. Authors interpret these results and shed light on the influence of stochastic and deterministic processes on microbial assemblages and propose a conceptual model of how changes in paleoclimate influence ecological succession.

George et al., studied hot spring microbiomes from Sembawang Hot Spring in Singapore which revealed assemblages that were dominated by phototrophic bacteria. Microbial diversity was inversely correlated to environmental stress defined by temperature, sulfide, and carbonate concentrations. This article adds information for the characterization of hot spring microbiomes.

Savaglia et al., also characterized Antarctic microbial mats, focusing on the connections between habitat and eukaryotic and prokaryotic microbes in soils of the Sør Rondane Mountains. Even in these extreme habitats, the authors were able to show differentiation between community composition in different soil types and stressed the role of lithology in supporting microbial diversity.

In extreme habitats microbial communities dominate energy and nutrient dynamics. Stanish et al., working in streams of the McMurdo Dry Valleys, used an elegant approach to understanding the contributions of different types of microbial mats to particulate organic matter flux using the relative abundance of indicator diatoms. They were able to infer the varying importance to organic matter export of these mat types, which occupied different stream sub-habitats, over daily to seasonal timescales and stream discharge dynamics.

Acosta et al. describe the protist communities living within microbial mats in high elevation lagoons in the Atacama. They use metabarcoding to show the diversity of eukaryotes, using both DNA and cDNA approaches that allowed both the presence and the activity of different taxa to be inferred. The authors note that salinity is a key predictor of the active elements of the microbiota, and infer that mat communities contain a mix of active and resting organisms that are able to switch on and off in response to changing water salinity.

Lumian, Grettenberger et al., presented four new Antarctic cyanobacteria metagenome-assembled genomes (MAGs) from microbial mats in Lake Vanda in the McMurdo Dry Valleys in Antarctica. Their results showed that these cyanobacteria have genes coding for various cold tolerance mechanisms and most standard circadian rhythm genes. This study demonstrated that polar cyanobacteria genomes are underrepresented in reference databases and there is continued need for genome sequencing of polar cyanobacteria.

Lumian, Sumner et al., also contribute to our current knowledge of cyanobacterial geographic distribution patterns in Antarctica with an interesting approach consisting of using large-scale k-mer searches in metagenome-assembled genomes. Authors assign five cyanobacteria from Lakes Fryxell and Vanda that have distinct distribution patterns ranging from locally restricted to globally occurring.

This special edition reinforces the importance of microbial mats as a fundamental component of the biodiversity of extreme environments. The papers demonstrate remarkable similarities between the composition of microbial mats from a range of extreme habitats and the ability of a sufficient functional diversity of microbes to evolve extremophilic physiologies to create sustainable communities. A feature of the papers presented here is the need to recognize that even within environments recognized as extreme for a specific factor, such as heat or cold, dominant microbes were selected by other environmental variables (such as salinity) indicating perhaps that competitive interactions may be important even in the most extreme habitats. Dominance by eubacteria in many functions was evident, but the need to recognize the diversity of eukaryotes that are supported by microbial mats, and their contributions to productivity, was evident. The importance of microbial mats as oases of diversity and productivity in environmentally challenging locations is now widely accepted, but it is clear that there is still much research needed to understand the nuances of these remarkable communities.

## Author contributions

IH: Conceptualization, Writing – original draft, Writing – review & editing. JG-M: Conceptualization, Writing – original draft, Writing – review & editing. LF: Conceptualization, Writing – original draft, Writing – review & editing.

